# A rare case of malignant giant phyllodes tumour with retrosternal extension and pericardial invasion

**DOI:** 10.1259/bjrcr.20150357

**Published:** 2016-05-15

**Authors:** Rishi Philip Mathew, Ram Shenoy Basti, Hadihally B Suresh

**Affiliations:** Department of Radiodiagnosis, Father Muller Medical College, Mangalore, India

## Abstract

Phyllodes tumour was first described in 1838 by Johannes Muller. These tumours are uncommon and comprise < 0.5% of all breast neoplasms. Among the three histological subtypes—benign, borderline and malignant—the malignant variety is the most uncommon. Giant phyllodes tumours measure > 10 cm in their largest dimension. Overall prognosis for these lesions is poor, with high recurrence rates. Surgery with post-operative adjuvant chemoradiotherapy is the main treatment for malignant giant phyllodes tumours. We present a rare case of malignant giant phyllodes tumour of the left breast in a 23-year-old female patient with retrosternal extension and invasion of the pericardium.

## Summary

Phyllodes tumour was first described in 1838 by Johannes Muller. These tumours are uncommon and comprise < 0.5% of all breast neoplasms. Among the three histological subtypes—benign, borderline and malignant—the malignant variety is the most uncommon. Giant phyllodes tumours measure > 10 cm in their largest dimension. Overall prognosis for these lesions is poor, with high recurrence rates. Surgery with post-operative adjuvant chemoradiotherapy is the main treatment for malignant giant phyllodes tumours. We present a rare case of malignant giant phyllodes tumour of the left breast in a 23-year-old female patient with retrosternal extension and invasion of the pericardium.

## Clinical presentation

**Figure 1.**
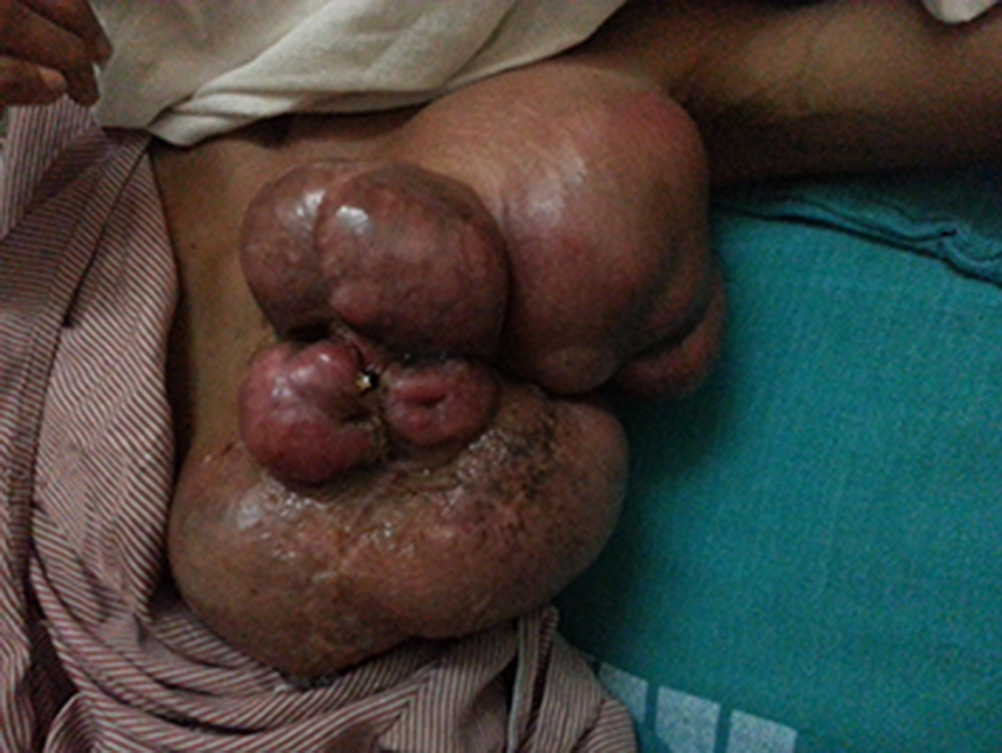
Malignant giant phyllodes tumour of the left breast in a 23-year-old female patient.

A 23-year-old female patient presented to the oncosurgery department with complaints of a large left breast mass for 3 years (Figure 1). The mass initially presented as a small lump, which gradually grew in size. During this time, she felt no pain or discomfort. In the past few months, the patient noticed rapid growth in the size of the lump along with discolouration of the breast skin.

Clinical examination revealed an enlarged breast completely replaced by a lobulated mass that measured approximately 21 × 12 × 13 cm. No enlarged axillary lymph nodes were noted. The contralateral right breast was normal in appearance as well as on palpation.

**Figure 2.**
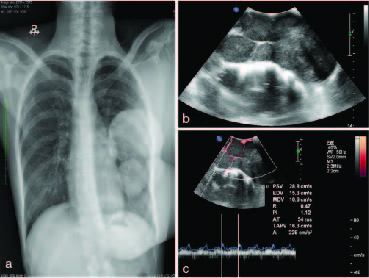
(a) Chest radiograph showing a large multilobulated soft tissue density lesion involving the left breast with normal bilateral lung fields and bony thoracic cage. (b) Ultrasound of the left breast revealing a circumscribed multilobulated solid heteroechoic lesion with internal vascularity on (c) colour Doppler. AI, average intensity; AT, average time; EDV, end-diastolic velocity; MDV, mean diastolic velocity; PI, pulsatility index; PSV, peak systolic velocity; PW, pulsed wave; RI, resistive index; SV, stroke volume; TAPV, time-averaged peak velocity.

**Figure 3.**
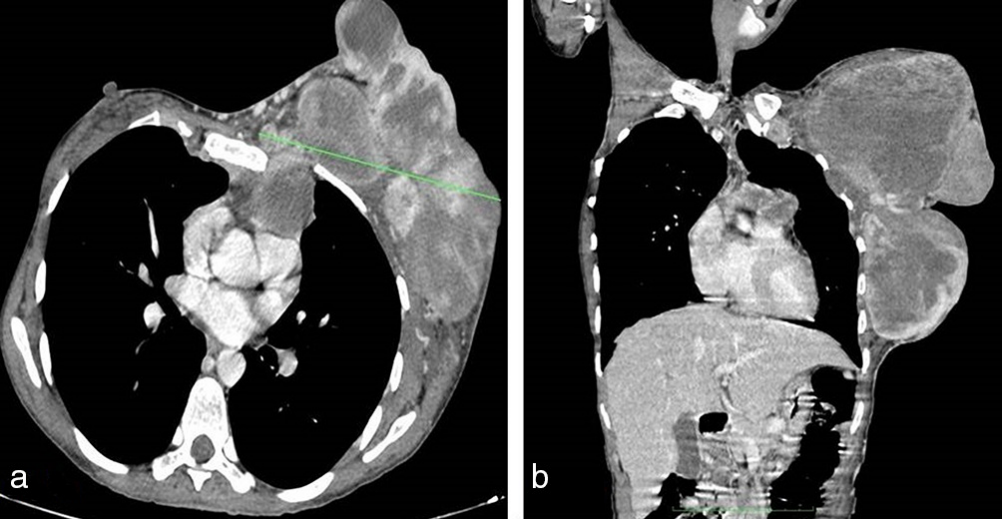
Post-contrast CT scan of the chest. (a) Axial and (b) reformatted coronal images showing a heterogeneously enhancing left breast mass with central necrosis. The lesion showed erosion of the sternum with retrosternal extension to involve and invade the pericardium.

**Figure 4.**
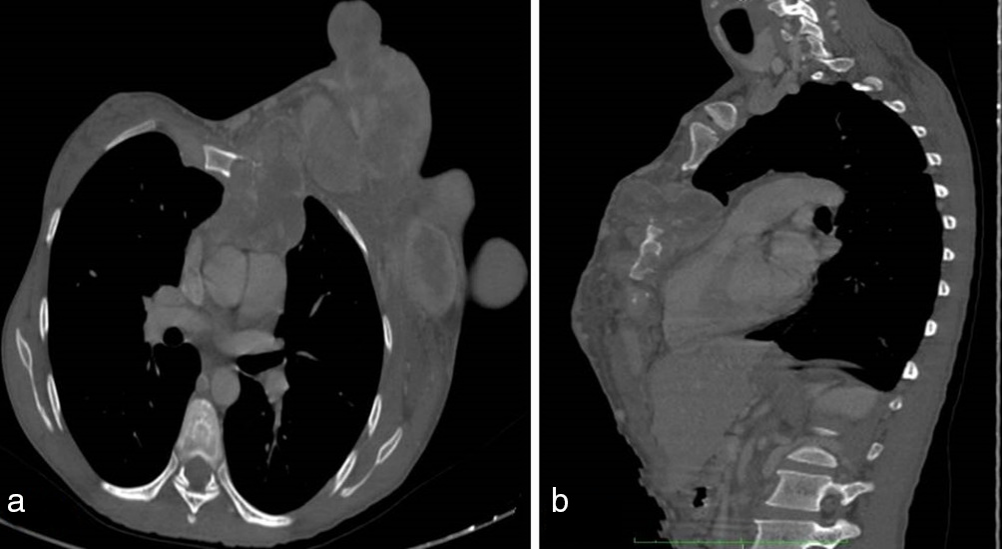
CT scan (a) axial and (b) reformatted sagittal bone window images showing erosion of the sternum.

Initial chest radiograph (Figure 2a) showed a multilobulated soft tissue density lesion involving the left breast. Bilateral lung fields and the bony thoracic cage appeared normal. On ultrasound (Figure 2b), the lesion appeared as a circumscribed, multilobulated, heteroechoic solid lesion, with internal vascularity on colour Doppler (Figure 2c). As the lesion was massive in size and could not be adequately assessed by radiography or ultrasound, the patient was referred for CT scan of the chest for further evaluation. Post-contrast CT scan of the chest (Figures 3 and 4) revealed a heterogeneously enhancing aggressive lesion measuring 21 × 15 × 13 cm with central areas of necrosis, with evidence of sternal erosion and invasion of the pericardium. The adjacent vessels, lungs and cardia appeared normal with no evidence of invasion. There was no evidence of lymphadenopathy. The patient underwent left mastectomy followed by adjuvant chemoradiotherapy. Histopathological evaluation of the excised specimen confirmed the diagnosis of malignant phyllodes tumour.

## Discussion

Phyllodes tumour was first described by Johannes Muller in 1838. It is an uncommon neoplasm of the breast with an incidence of 1 per 100,000 and accounts for about 0.5% of all breast tumours. Although these tumours are sometimes known as cystosarcoma phyllodes, the term is best avoided as most of these tumours turn out to be benign.^1^ According to their histopathology, they may be classified as benign, malignant or borderline.^2^ The malignant variety most commonly affects females aged between the fourth and sixth decades and its incidence is less than the benign variety of phyllodes.^3^ Clinically, both the benign and malignant varieties cannot be differentiated as they often appear as a mobile, round and non-tender mass.^4^ Giant phyllodes tumours are defined as tumours that measure > 10 cm in their largest dimension. Imaging features of giant phyllodes tumours have rarely been reported owing to its extremely low incidence. On mammograms, these lesions may present as isodense masses with plaque-like calcifications. Chung et al^5^ evaluated 14 cases of giant phyllodes tumours and found these lesions to be circumscribed, lobulated and heterogeneous in echotexture on ultrasound. Some of the lesions may have a cystic component. A CT scan is useful in evaluating large lesions that cannot be assessed by mammography, ultrasound or chest radiography, as well as the lymph nodal status. Chung et al^5^ also noticed that the size of the tumour showed a linear relationship with histological grading, with larger tumours being malignant. On dynamic MRI, some of these lesions showed an initial rapid and delayed persistent enhancement pattern, with internal signal voids. These features may be useful in differentiating giant phyllodes tumours from giant fibroadenomas, which are often difficult to differentiate both on imaging and histology. Positron emission tomography may be useful in differentiating a benign phyllodes tumour from a malignant one, with the latter often having a standardized uptake value < 2.5. Fine needle aspiration cytology is avoided for these tumours as it tends to be unhelpful, and even core biopsy only has a moderate sensitivity at best; therefore, surgical resection is often the only way of determining whether a lesion is malignant or not.^5,6^ Irrespective of the histological subtype, surgery with an excision margin of 1–2 cm is the main treatment of choice, as recurrences tend to be more common in patients with an excision margin of < 1–2 cm.^7^ Involvement of lymph nodes is uncommon in phyllodes tumour and hence routine axillary lymph node dissection can be avoided. However, removal of axillary lymph nodes may be warranted in patients with a palpable lymph node or those presenting with a large mass.^8,9^ Although uncommon, malignant phyllodes tumours can metastasize, most commonly to the lungs, mediastinum and bones, and rarely to the liver. The prognosis for malignant phyllodes tumours is poor, while giant phyllodes tends to show high recurrence (41%) and cancer rates (42.5%). Hence, these lesions require more aggressive management, including multimodality post-operative treatment such as adjuvant radiotherapy and chemotherapy.^10^

## Learning points

1. Giant phyllodes tumour is defined as a tumour that measures > 10 cm in the largest dimension.
2. Phyllodes tumours are histologically classified into benign, borderline and malignant.
3. The malignant variety is the most uncommon among the three and shows a high recurrence rate.
4. Clinically, imaging-wise and histologically, it may be difficult to differentiate a giant phyllodes tumour from a giant fibroadenoma, which is the main differential diagnosis.
5. On radiographs, these lesions appear as soft tissue density lesions with or without plaque-like calcification. On ultrasound, they appear as large, circumscribed, multilobulated, heteroechoic lesions with or without cystic components. Dynamic MRI may be useful in differentiating phyllodes tumours from other lesions, as the former shows an initial rapid and delayed persistent enhancement. Positron emission tomography may help in differentiating benign from malignant phyllodes, as the latter has a standardized uptake value < 2.5, which suggests malignancy.
6. Surgery with postoperative adjuvant chemoradiotherapy is the mainstay of treatment for malignant giant phyllodes tumour.

## Consent

Written informed consent was obtained from the patient for publication of this case report, including the accompanying images.
